# Chronic Disease Prevalence in the US: Sociodemographic and Geographic Variations by Zip Code Tabulation Area

**DOI:** 10.5888/pcd21.230267

**Published:** 2024-02-29

**Authors:** Gabriel A. Benavidez, Whitney E. Zahnd, Peiyin Hung, Jan M. Eberth

**Affiliations:** 1 Baylor University, Department of Public Health, Waco, Texas; 2 University of Iowa, Department of Health Management and Policy, Iowa City, Iowa; 3 University of South Carolina, Department of Health Services Policy and Management, Columbia, South Carolina; 4 Drexel University, Department of Health Management and Policy, Philadelphia, Pennsylvania

## Abstract

**Introduction:**

We examined the geographic distribution and sociodemographic and economic characteristics of chronic disease prevalence in the US. Understanding disease prevalence and its impact on communities is crucial for effective public health interventions.

**Methods:**

Data came from the American Community Survey, the American Hospital Association Survey, and the Centers for Disease Control and Prevention’s PLACES. We used quartile thresholds for 10 chronic diseases to assess chronic disease prevalence by Zip Code Tabulation Areas (ZCTAs). ZCTAs were scored from 0 to 20 based on their chronic disease prevalence quartile. Three prevalence categories were established: least prevalent (score ≤6), moderately prevalent (score 7–13), and highest prevalence (score ≥14). Community characteristics were compared across categories and spatial analyses to identify clusters of ZCTAs with high disease prevalence.

**Results:**

Our study showed a high prevalence of chronic disease in the southeastern region of the US. Populations in ZCTAs with the highest prevalence showed significantly greater socioeconomic disadvantages (ie, lower household income, lower home value, lower educational attainment, and higher uninsured rates) and barriers to health care access (lower percentage of car ownership and longer travel distances to hospital-based intensive care units, emergency departments, federally qualified health centers, and pharmacies) compared with ZCTAs with the lowest prevalence.

**Conclusion:**

Socioeconomic disparities and health care access should be addressed in communities with high chronic disease prevalence. Carefully directed resource allocation and interventions are necessary to reduce the effects of chronic disease on these communities. Policy makers and clinicians should prioritize efforts to reduce chronic disease prevalence and improve the overall health and well-being of affected communities throughout the US.

**Summary**

**What is known on this topic?**

Chronic diseases are increasing in prevalence throughout the US and put a major strain on the health care system.

**What this study adds**

Areas affected by a high prevalence of multiple chronic diseases face a variety of socioeconomic and environmental barriers to achieving good health. Many risk factors for chronic disease are likely beyond the individual’s control and require large-scale policy change.

**What are the implications for public health practice?**

Preventing and managing chronic disease will require combatting poverty and socioeconomic inequality.

## Introduction

An estimated 129 million people in the US have at least 1 major chronic disease (1) (eg, heart disease, cancer, diabetes, obesity, hypertension) as defined by the US Department of Health and Human Services (2). Five of the top 10 leading causes of death in the US are, or are strongly associated with, preventable and treatable chronic diseases (3). Over the past 2 decades prevalence has increased steadily, and this trend is expected to continue (4). An increasing proportion of people in America are dealing with multiple chronic conditions; 42% have 2 or more, and 12% have at least 5 (5). Besides the personal impact, chronic disease has a substantial effect on the US health care system. About 90% of the annual $4.1 trillion health care expenditure is attributed to managing and treating chronic diseases and mental health conditions (6).

The increasing focus on studying social determinants of health (SDOH) has generated several studies showing a connection between chronic disease and socioeconomic factors. One study found a significantly lower prevalence of asthma, arthritis, diabetes, hypertension, and obesity in affluent counties compared with the least affluent ones (7). Another study showed that people with less than a high school education had nearly twice the odds of having diabetes compared with those with a college degree (8). Across large US cities, rates of stroke and hypertension are concentrated in census tracts with a high proportion of Black residents, old homes with low market value, and people receiving government assistance (9).

Although many chronic diseases are partly the result of individual health behaviors, such as excessive drinking, smoking, sedentary lifestyles, and poor nutrition, structural socioeconomic and environmental factors play a role in their development, prevention, and management. Although poor nutrition may appear to be an individual choice, affordability and convenient access to high quality, nutritious food are often the primary determinants of food choice for people with a low income (10). Similarly, leisure-time physical activity is often framed as a simple behavior that people should incorporate into their daily lives. However, this framing puts the onus on the individual and fails to recognize time, financial, and built environment factors (eg, safe neighborhood, access to parks) that influence a person’s ability to engage in regular physical activity (11). Once a chronic condition develops, management of the disease requires the ability to afford health care and have physical access to it. Yet research increasingly shows that even when medically necessary, people often forgo costly medications and health care, hindering their ability to manage their disease (12,13).

Although the literature examining both area-level and individual-level chronic disease and SDOH is expansive, current literature has 3 major shortcomings: 1) on a national scale, work is often limited to county-level analyses, 2) most current research focuses on individual chronic diseases and their association with SDOH, and 3) studies often include metrics of only socioeconomic status, such as income or educational attainment, as an SDOH measure. These shortcomings are notable because of the potential for wide variation in chronic disease prevalence and SDOH factors within counties. An average US county population is over 100,000, but smaller geographic units, such as Zip Code Tabulation Areas (ZCTAs), which are generalized areal representations of the geographic coverage and distribution of the zip codes, have an average population of less than 10,000. One study of the Chicago, Illinois, area (14) aimed to fill some of this research gap by examining multiple dimensions of SDOH in relation to premature death at the census tract level and found that all their SDOH dimensions (eg, socioeconomic position, housing, transportation, physical disability) were associated with higher premature death. That study highlighted the growing interest among researchers in examining the multidimensional factors of SDOH and their effect on health outcomes. However, it was limited in scope because it examined 1 health outcome metric, that is, premature death, and in only 1 city.

To add to the body of literature and address the shortcomings of previous research, our study had 3 aims: 1) to create a community indicator of chronic disease prevalence at a subcounty level (ie, areas with high or low prevalence of multiple chronic diseases) by using ZCTAs, 2) to examine the geographic distribution of chronic disease prevalence, and 3) to examine the differences in socioeconomic and environmental characteristics of ZCTAs with low and high chronic disease prevalence.

## Methods

### Data sources

Data on ZCTA-level chronic disease prevalence estimates came from the 2020 Centers for Disease Control and Prevention’s (CDC’s) PLACES data set. The PLACES project is designed to generate health data for small geographic units across the country (ie, county, census tract, and ZCTA) to enable informed decisions when planning public health interventions. The PLACES estimates are derived by using small-area estimation methodologies along with health-indicator data from the Behavioral Risk Factor Surveillance System (BRFSS) survey (15) adapted by each state, which were originally designed to produce valid state-level prevalence estimates. To produce estimates at the ZCTA level, CDC uses a multivariable approach that incorporates demographic and socioeconomic data. This generates reliable estimates of chronic disease prevalence for smaller geographic units. Details of the PLACES methodology are described elsewhere (16).

ZCTA-level sociodemographic and economic data were obtained from the American Community Survey (17). As recommended when examining small geographic units, we used 5-year (2015–2019) estimates for all included variables. Additionally, PLACES 2020 data estimates are derived mainly from the 2019 BRFSS but also include the 2018 BRFSS for some modules of questions that are not asked every year. We also obtained data on availability of health care services (hospital-based intensive care units [ICUs], emergency departments [EDs], federally qualified health centers [FQHCs], and pharmacies) from the 2019 American Hospital Association Annual Survey (18), the Center for Medicare and Medicaid Services Provider of Service Files (19), and the US Department of Agriculture Service Area Map Datasets’ Healthcare facilities file (20). Our analysis included 31,634 of the approximately 32,000 ZCTAs in the US. PLACES excludes ZCTAs with fewer than 50 people.

### Chronic disease prevalence score

As an indicator of chronic disease prevalence, we created a composite score that allowed us to identify ZCTAs estimated to have a high prevalence of multiple chronic diseases. We used the 10 most prevalent and costly chronic diseases in the US (21): obesity, hypertension, high cholesterol, coronary heart disease, chronic obstructive pulmonary disease, asthma, chronic kidney disease, diabetes, cancer (excluding skin cancer), and depression. For each disease, we ordered all ZCTAs by disease prevalence and assigned a score of 0 to ZCTAs with a chronic disease prevalence in the bottom 25th percentile, 1 for ZCTAs in the middle (between the 25th and the 75th percentiles), or 2 to those in the top 25th percentile for each chronic disease (Table 1). We summed the scores for each chronic disease included together to provide a total score ranging from 0 to 20. A score of 0 indicated that the ZCTA was in the lowest 25th percentile for all 10 disease prevalence estimates, and those with a score of 20 were in the top 25th percentile for all 10 disease prevalence estimates. We then created 3 chronic disease prevalence categories by using quartile thresholds as before (in the bottom 25th percentile, between the 25th and 75th percentiles, and in the top 25th percentile): the lowest prevalence ZCTAs (score ≤6), moderate prevalence ZCTAs (score of 7–13), and highest prevalence ZCTAs (score ≥14).

**Table 1:** Chronic Disease Prevalence Estimate Thresholds by Percentile^a^

Chronic condition	Modeled ZCTA prevalence estimates, percentile		
	Bottom 25%	Middle 50%	Top 25%
Obesity	31.2	>31.2–<37.8	37.8
Diabetes	9.4	>9.4–<13.1	13.1
Hypertension	30.6	>30.6–<38.8	38.8
High cholesterol	31.3	>31.3–<36.6	36.6
Coronary heart disease	5.7	>5.7–<8.2	8.2
Chronic obstructive pulmonary disease	6.4	>6.4–<9.8	9.8
Asthma	9.1	>9.1–<10.6	10.6
Chronic kidney disease	2.7	>2.7–<3.5	3.5
Cancer (excluding skin cancer)	6.7	>6.7–<8.4	8.4
Depression	19.3	>19.3–<23.9	23.9

Abbreviation: ZCTA, zip code tabulation area.

a Data are from the 2020 Population Level Analysis of Community Estimates (PLACES) by Centers for Disease Control and Prevention (16). The prevalence distribution of each chronic condition across ZCTAs was used to categorize the quartile distributions and further categorize ZCTAs into 3 groups: the bottom 25th percentile (those with the lowest prevalence), the middle 50% (between the 25th and the 75th percentiles), or the top 25th percentile for each chronic disease (those with the highest prevalence).

### Covariates

**Figure 1:**
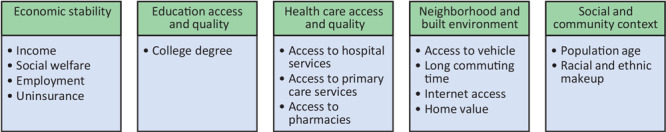
Categories of variables included, based on Healthy People 2030 Social Determinants of Health framework (22), in study of sociodemographic and geographic variations of chronic disease prevalence in the US by Zip Code Tabulation Area.

**Sociodemographic and economic status, distance to health care services.** We included variables in this analysis that encompassed multiple aspects of the Healthy People 2030 Social Determinants of Health domains (Figure 1) (22). By using data from the 2019 American Community Survey’s 5-year estimates (2015–2019) (17) we captured ZCTA-level characteristics that included economic status, defined as median income, median home value, and proportion of residents receiving Supplemental Nutrition Assistance Program (SNAP); racial and ethnic characteristics; access to health care (eg, proportion without health insurance); travel barriers (eg, proportion without a vehicle, proportion with >1 h employment commute); and proxies of social disadvantage (eg, proportion of residents elderly, in households without medical insurance, foreign born).

All the health care service files we used provided physical addresses of the facilities that were being examined. We used the World Geocoding Service from ArcGIS Pro (Esri) to perform batch geocoding of facilities for all health care services included in our analysis. We used the GEODIST function in SAS version 9.4 (SAS) to calculate the distance in miles from each residential population-weighted ZCTA centroid to the nearest health care facility for all facilities included in our analysis (ICUs, EDs, FQHCs, and pharmacies).

**Analysis.** To examine the geographic distribution of chronic disease, we created choropleth maps of prevalence scores in ArcGIS Pro. We then performed a hot spot analysis, also in ArcGIS Pro, to see where ZCTAs with either high or low prevalence were clustered together. The hot spot analysis tool in ArcGIS Pro calculated the Getis-Ord Gi* statistic for each ZCTA in the analysis. A positive Gi* statistic indicated intensity of clustering around high values, and a negative Gi* statistic indicated clustering of low values. We also applied the default ArcGIS Pro false discovery rate correction to account for multiple testing and spatial dependency in the hot spot analysis.

By using the Kolmogorov–Smirnov goodness of fit test we determined that the use of the Kruskal–Wallis test — a nonparametric hypothesis testing — was most appropriate for our data. To further examine specific differences between chronic disease prevalence groups, we then used the Dwass, Steel, Critchlow-Fligner multiple comparison test to perform pairwise comparisons of groups. Of specific interest was determining whether there was a significant difference in the characteristics of ZCTAs with the highest and lowest chronic disease prevalence. A 2-tailed *P* value of <.05 was used to determine significance in all hypothesis testing.

## Results

**Figure 2:**
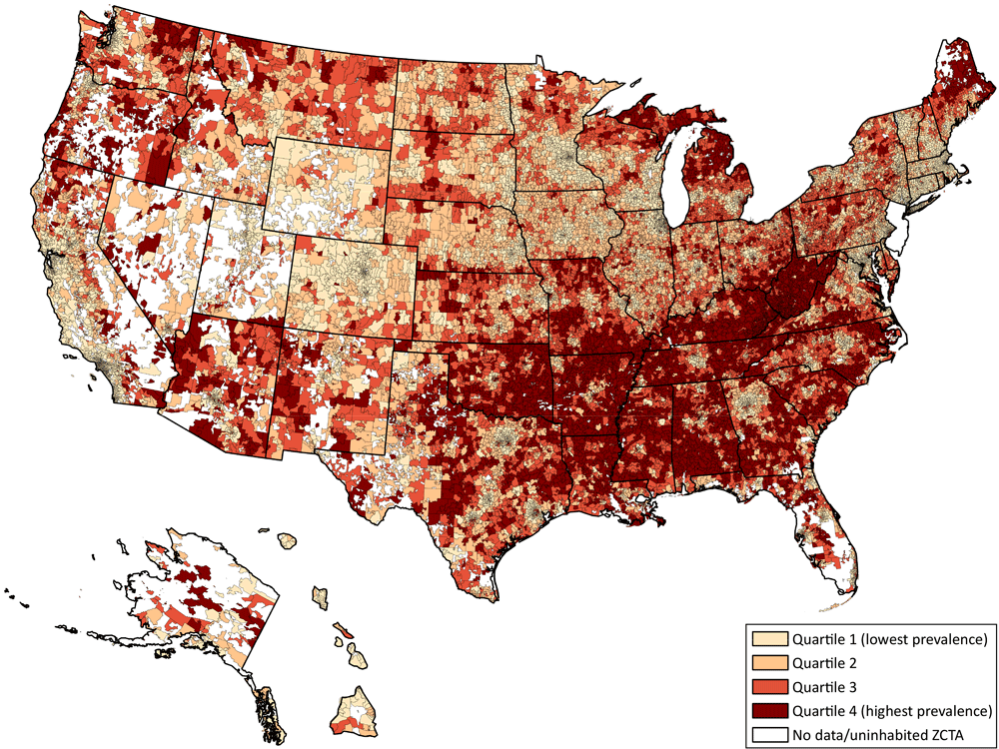
Choropleth map of the US showing the geographic distribution of chronic disease prevalence scores by quartile across Zip Code Tabulation Areas (ZCTAs). Chronic disease prevalence scores ranged from 0 to 20 with a score of 0 meaning the ZCTA was in the 25^th^ percentile and a score of 20 meaning the ZCTA was in the 75^th^ percentile of prevalence for each chronic disease examined.

**Figure 3:**
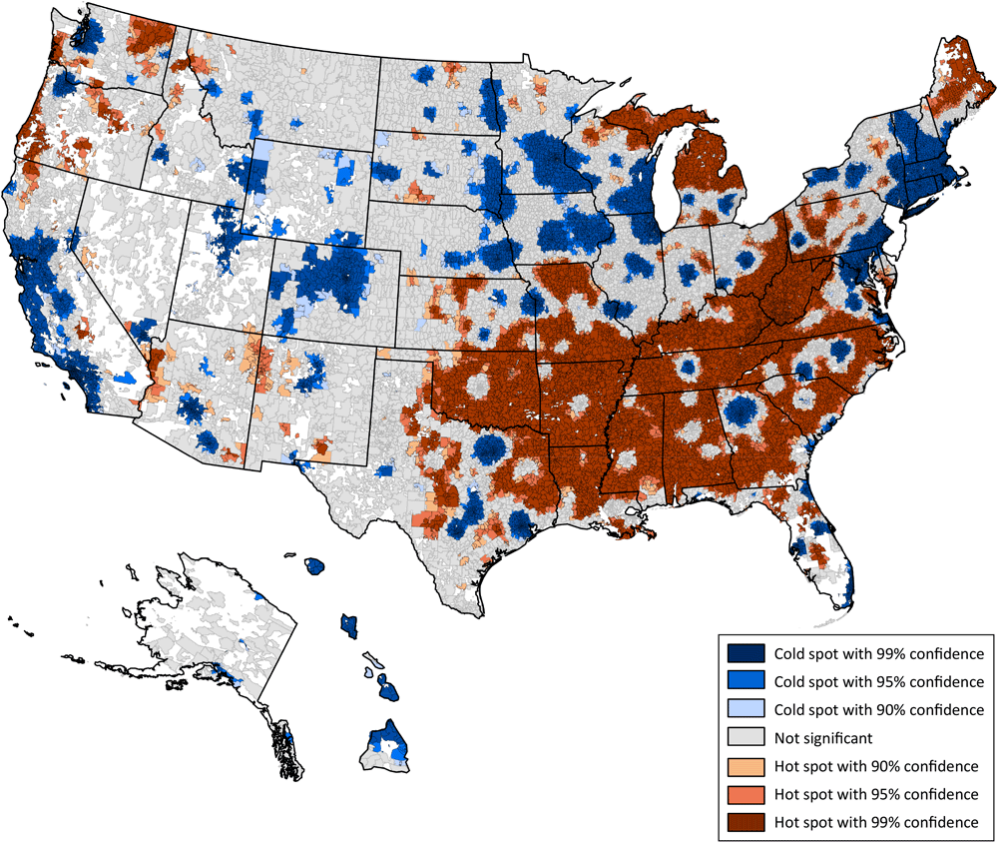
Hot Spot analysis of chronic disease prevalence scores throughout the US calculated in ArcGIS Pro (Esri) showing significant spatial clusters of high chronic disease prevalence scores (red clusters = hot spots) and low chronic disease prevalence scores (blue clusters = cold spots).

Of the 31,634 ZCTAs included in our study, 8,576 (27.1%) fell into the least prevalent (Quartile 1) category, 14,670 (46.4%) into the moderate prevalence category (Quartiles 2 and 3), and 8,388 (26.5%) into the highest prevalence category (Quartile 4) (Figure 2). The hot spot analysis (Figure 3) showed that high chronic disease scores (hot spots) were predominantly clustered throughout the southeastern region of the US, with additional high score clustering in parts of Maine, Michigan, and the Pacific Northwest. Less apparent patterns emerged when examining clusters of low chronic disease scores (cold spots) because they are disbursed throughout the US but appear to cluster around major cities. For example, in Texas, the 3 cold spot clusters in the eastern part of the state are in the metropolitan areas of Dallas, Houston, Austin, and San Antonio. The largest cold spot in the state of Georgia is the area surrounding the Atlanta metropolitan region.

Differences in ZCTA characteristic median values across all categories of chronic disease prevalence were significant (*P* < .05) for all included variables (Table 2). ZCTAs in the lowest prevalence category had higher population sizes (median, n = 12,955) than those in the moderate category (median, n = 2,270) or highest category (median, n = 1,398). ZCTAs in the lowest prevalence category had populations that were younger (median age, 38.4 y; interquartile range [IQR], 34.2–43.1 y) compared with the moderate category (median age, 42.6; y IQR, 37.8–47.7 y) or highest category (median age, 44.4 y; IQR, 39.3–51.2 y).

**Table 2:** Characteristics of Zip Code Tabulation Areas by Category of Chronic Disease Prevalence

Characteristic	Lowest prevalence (n = 8,576)	Moderate prevalence (n = 14,670)	Highest prevalence (n = 8,388)	*P* value	Low vs high *P* value^a^
	Median (IQR)	Median (IQR)	Median (IQR)		
**Demographics**					
Population size, no.	12,955 (2501–29923)	2,270 (689–9118)	1,398 (453–4356)	<.001	<.001
Median age, y	38.4 (34.2–43.1)	42.6 (37.8–47.7)	44.4 (39.3–51.2)	<.001	<.001
Aged ≥65 y, %	13.8 (10.3–17.3)	18.1 (14.5–22.4)	20.2 (16.15–26.2)	<.001	<.001
Foreign-born, %	8.8 (3.8–17.9)	2.5 (0.87–6.1)	1.3 (0–3.2)	<.001	<.001
Have a disability, %	11.4 (7.9–12.1)	14.54 (11.8–17.6)	19.6 (15.9–24.3)	<.001	<.001
Uninsured, %	5.3 (3.0–8.9)	7.2 (4.2–11.9)	9.8 (5.7–15.0)	<.001	<.001
Unemployed, %	2.8 (1.9–3.8)	2.8 (1.5–4.2)	3.2 (1.5–5.1)	<.001	.008
Have internet, %	86.3 (80.5–90.8)	74.0 (68.9–81.1)	66.9 (57.3–73.2)	<.001	<.001
Median income, $	73,929 (60,250–93,400)	53,851 (46,157–62,857)	40,750 (34,200–47,917)	<.001	<.001
Median home value, $	257,700 (180,200–393,000)	133,300 (98,000–177,800)	91,600 (72,800–121,500)	<.001	<.001
Receive SNAP, %	6.4 (3.4–10.8)	10.7 (6.21–16.3	16.9 (10.2–24.1)	<.001	<.001
Post high school education, %	68.1 (57.9–78.5)	52.2 (43.8–60.4)	42.9 (35.3–50.1)	<.001	<.001
No personal vehicle, %	3.5 (1.7–6.3)	3.9 (1.7–6.9)	5.6 (2.3–9.7)	<.001	<.001
More than 1-hour work commute, %	6.4 (3.3–12.4)	6.6 (3.45–10.8)	7.6 (3.8–13.7)	<.001	.004
Race or ethnicity, %					
American Indian/Alaska Native	0.7 (0.1–1.1)	1.5 (0.9–1.8)	2.7 (1.7–3.0)	<.001	<.001
Asian	5.7 (3.4–6.8)	0.9 (0.6–1.1)	0.5 (0.2–0.8)	<.001	<.001
Black	6.6 (3.9–7.1)	5.9 (3.8–7.3)	11.9 (9.4–14.6)	<.001	<.001
Hispanic	13.9 (10.8–16.7)	8.8 (4.3–11.2)	4.8 (2.6–6.3)	<.001	<.001
White	89.5 (71.3–94.4)	94.7 (85.2–98.0)	92.7 (71.2–98.0)	<.001	.10
Distance to nearest health care services, miles					
Nearest FQHC	4.6 (1.8–9.6)	9.4 (3.8–17.3)	8.7 (3.1–14.8)	<.001	<.001
Nearest ICU	5.6 (2.6–10.8)	12.8 (6.6–20.8)	18.2 (10.9–27.4)	<.001	<.001
Nearest ED	5.3 (2.4–9.8)	10.6 (5.5–16.5)	14.6 (8.7–21.8)	<.001	<.001
Nearest pharmacy	0.9 (0.4–3.3)	4.3 (0.86–8.6)	5.7 (1.3–10.1)	<.001	<.001

Abbreviations: ED, emergency department; FQHC, federally qualified health center; ICU, intensive care unit; IQR, interquartile range; SNAP, Supplemental Nutrition Assistance Program.

a Pairwise comparison comparing the highest chronic disease burden group to the lowest chronic disease burden group.

Pairwise comparisons to examine differences in characteristics between ZCTAs with the highest and lowest chronic disease prevalence also showed significant differences for all sociodemographic and economic factors. Compared with ZCTAs with the lowest prevalence, those with the highest prevalence of chronic disease had significantly higher proportions of people with a disability (19.6% vs 11.4%; *P* <.001) and who were uninsured (9.8% vs 5.3%; *P* <.001), unemployed (3.2% vs 2.8%; *P* =.008), received SNAP benefits (16.9% vs 6.4%; *P* <.001), had no personal vehicle (5.6% vs 3.5%; *P* <.001), or had to commute more than 1 hour for work (7.6% vs 6.4%; *P* = .004). ZCTAs in the lowest prevalence category also had significantly higher proportions of residents with post–high school education (68.1% vs 42.9%; *P *<.001) who were living in a home with internet access (83.0% vs 66.9%; *P* <.001) and had higher median incomes ($73,929 vs $40,750; *P* <.001) and higher home values ($257,000 vs $91,600; *P* <.001) than those in ZCTAs with the highest prevalence.

Racial demographics also differed. ZCTAs in the highest chronic disease prevalence category had a significantly higher proportion of Black residents (11.9% vs 6.6%, *P* <.001) and American Indian/Alaska Native residents (2.7% vs 0.7%, *P* <.001) compared with the lowest prevalence ZCTAs. In contrast, the proportions of Asian residents (5.7% vs 0.5%, *P* <. 001) and Hispanic (13.9% vs 4.8%, *P* <.001) residents were significantly lower in ZCTAs with the highest prevalence than in those with the lowest prevalence. The proportion of White residents (89.5% vs 92.7.5%, *P* = .10) did not differ significantly between ZCTAs with the highest and lowest prevalence.

Residents of ZCTAs with the lowest chronic disease prevalence lived significantly closer to health care services than those in ZCTAs with the highest prevalence (*P* <.001). The median distance to the nearest FQHC for ZCTAs with the lowest chronic disease prevalence was 4.6 miles (IQR, 1.8–9.6 miles), whereas it was 8.7 miles (IQR, 3.1–14.8 miles) for residents of ZCTAs with the highest prevalence. The nearest hospital services were also closer for residents of ZCTAs with the lowest prevalence (ICU [median = 5.6 miles; IQR, 2.6–10.8]; ED [median = 5.3 miles, IQR, 2.4–9.8 miles]) than for those with the highest prevalence (ICU [median = 18.2 miles; IQR, 10.9–27.4]; ED: [median = 14.6 miles; IQR, 8.7–21.8 miles]). Although pharmacies were closer than any of the health care facilities in all 3 prevalence categories, the median distance was less than a mile for the lowest prevalence ZCTAs compared with the highest (5.7 miles, IQR, 1.3–10.1 miles).

## Discussion

Our study created a composite score for chronic disease prevalence that included the 10 most prevalent and costly diseases. This allowed us to analyze the nationwide distribution of chronic disease and identify regions with the highest prevalence. Additionally, we explored variations in community characteristics across the spectrum of chronic disease prevalence. Prevalence was highest in the southeastern US, whereas areas with low prevalence were more geographically diverse, spanning large metropolitan areas nationwide. Compared with areas with the lowest prevalence, areas with the highest were significantly smaller in population size and had older, more socioeconomically disadvantaged residents and a higher proportion of Black or American Indian/Alaska Native residents. Residents in communities with the highest prevalence had to travel substantially longer distances for health care services than people in areas with the lowest prevalence.

Our results on the geographic distribution of chronic disease prevalence were consistent with previous work that focused on individual chronic diseases (7–10), but we also provide further evidence of the structural inequities in the southeastern region of the US. That region has consistently shown higher illness and death from chronic disease than other US regions (23). No single mechanism causes the elevated prevalence of disease in the southeastern region. The high prevalence of morbidity and mortality in this region is likely related to decades of social and economic policies (eg, an extremely low minimum wage, nonexpansion of Medicaid) that lag behind much of the rest of the country and have created environments conducive to concentrated poverty and lack of access to affordable health care (24). Our study showed that ZCTAs with the highest chronic disease prevalence had a high proportion of Black residents as does the South overall. Racism is prevalent throughout the US; however, the high proportion of Black residents in the South likely contributes to the major regional disparity in chronic disease prevalence. Black Americans continue to experience the trauma and negative consequences of chattel slavery and Jim Crow-era laws that have left generations worse off than their non-Black counterparts (24).

Sociodemographic and economic characteristics have been well studied and have shown consistently to be associated with various chronic diseases (25,26). Although our study corroborates these previous studies, we provide greater insight into the characteristics and the socioeconomic position of communities with a high prevalence of multiple chronic diseases. For example, our analysis showed that the median home price in areas of high prevalence was significantly lower than in neighborhoods with low prevalence. Previous research showed that neighborhood green and recreational space conducive to physical activity is strongly correlated with an area’s real estate values (27). Areas of high chronic disease prevalence and low real estate values likely lack community infrastructure conducive to physical activities and to interventions promoting it.

Effectively addressing chronic disease requires interventions and policy changes that consider the intricate interplay of socioeconomic and environmental factors and individual behavior choices. Unfortunately, most published literature on chronic disease interventions predominantly centers on modifying individual behavior for disease prevention and management (28). Some meta-analyses of commonly used intervention programs often find these interventions to have marginal effect sizes at best, even under the assumption of publication bias overestimating the program’s effectiveness (29). Although our study did not conclude that eliminating socioeconomic barriers to health care access would itself decrease chronic disease prevalence, the inseparable connection between those barriers and disease prevalence — at both the individual and community levels — requires careful consideration in the planning of public health interventions.

Our findings underscore disparities in health care access, particularly in communities with high chronic disease prevalence. Despite a higher need for health care services, residents in ZCTAs with the highest disease prevalence were 2 to 5 times farther from the nearest FQHCs, ICUs, EDs, and pharmacies than those in ZCTAs with the lowest prevalence. Combined with our finding that the greatest proportion of households without a vehicle were those with the highest prevalence of chronic disease, the inability to access health care for disease prevention and management may be a significant contributing factor to prevalence in these communities. In an analysis examining the changing landscape of hospital access in the South, Planey et al (30) noted the structural factors forcing hospital closures in rural, low-income, and census tracts with a high density of racial and ethnic minority residents, which result in longer travel distances for hospital care. That study, along with our findings, demonstrates the interconnectedness of structural and systemic inequities that contribute to disease prevalence among socioeconomically vulnerable populations. Other studies demonstrated how longer travel distances to access health care were associated with less frequent health care visits (31), lower rates of survival among people experiencing a cardiovascular event (32), and lower medication adherence (33), which all further contribute to poor disease prevention and management.

Our analysis provides a wholistic examination of multiple characteristics of communities dealing with high chronic disease prevalence by its use of a variety of well-validated and routinely used data sources. However, our study had limitations. First, the estimates of chronic disease prevalence were derived from BRFSS, which is designed to produce state- and county-level disease estimates. PLACES estimates are model-based and are generated by using multivariable regression models that incorporate BRFSS estimates of educational attainment and the county-level percentage of adults below 150% of the federal poverty level. Therefore, chronic disease prevalence estimates can be expected to correlate well with SDOH indicators, even outside those used to generate PLACES data. The size of some ZCTAs varies by geographic location, and this size difference may contribute to bias in some estimates of travel distances. However, we performed a stratified analysis based on ZCTAs’ rurality, and results remained consistent. By using ZCTAs as our unit of analysis, we could not examine several measures, such as health care service capacity or use volume. Such data were unavailable. These measures may have been a better indicator of health care service availability than our sole distance-based metric. However, distance-based measures at smaller geographic units are useful to provide a contextual understanding of the longer travel distances residents of an area experience, which is more difficult to conceptualize at the county level given the wide variation in the size of US counties.

### Conclusion

Chronic disease prevalence varies geographically and socioeconomically throughout the US. Residents of areas with the highest prevalence face social, economic, and environmental barriers that challenge prevention and management of the chronic disease. Public health efforts to combat the increasing prevalence of chronic disease will require large-scale interventions that focus on the complex interplay of behavioral and environmental factors to address the root causes of health disparities. Future public health interventions in chronic disease management and prevention should take into account nonbehavioral factors that influence the development and progression of chronic diseases. Continued failure to address the social and economic inequity throughout the US will likely result in the continued increase in chronic disease prevalence that disproportionately affects socioeconomically disadvantaged communities and people.

## References

[1] Boersma P , Black LI , Ward BW . Prevalence of multiple chronic conditions among US adults, 2018. *Prev Chronic Dis.* 2020;17:E106. 10.5888/pcd17.200130 32945769 PMC7553211

[2] Goodman RA , Posner SF , Huang ES , Parekh AK , Koh HK . Defining and measuring chronic conditions: imperatives for research, policy, program, and practice. *Prev Chronic Dis.* 2013;10:E66. 10.5888/pcd10.120239 23618546 PMC3652713

[3] Centers for Disease Control and Prevention. About chronic diseases. Published May 6, 2022. Accessed July 6, 2022. https://www.cdc.gov/chronicdisease/about/index.htm

[4] JAMA Network. The state of US health, 1990–2016. Burden of diseases, injuries, and risk factors among US states. Published 2018. Accessed July 6, 2022. https://jamanetwork.com/journals/jama/fullarticle/2678018 10.1001/jama.2018.0158 PMC5933332 29634829

[5] Buttorff C , Ruder T , Bauman M . Multiple chronic conditions in the United States. RAND Corporation; 2017. Accessed July 6, 2022. https://www.rand.org/pubs/tools/TL221.html

[6] Centers for Disease Control and Prevention. Health and economic costs of chronic diseases. Published June 6, 2022. Accessed July 6, 2022. https://www.cdc.gov/chronicdisease/about/costs/index.htm

[7] Shaw KM , Theis KA , Self-Brown S , Roblin DW , Barker L . Chronic disease disparities by county economic status and metropolitan classification, Behavioral Risk Factor Surveillance System, 2013. *Prev Chronic Dis.* 2016;13:E119. 10.5888/pcd13.160088 27584875 PMC5008860

[8] Borrell LN , Dallo FJ , White K . Education and diabetes in a racially and ethnically diverse population. *Am J Public Health.* 2006;96(9):1637–1642. 10.2105/AJPH.2005.072884 16873745 PMC1551936

[9] Fitzpatrick KM , Willis D . Chronic disease, the built environment, and unequal health risks in the 500 largest U.S. cities. *Int J Environ Res Public Health.* 2020;17(8):2961. 10.3390/ijerph17082961 32344643 PMC7215999

[10] Turrell G , Hewitt B , Patterson C , Oldenburg B , Gould T . Socioeconomic differences in food purchasing behaviour and suggested implications for diet-related health promotion. *J Hum Nutr Diet.* 2002;15(5):355–364. 10.1046/j.1365-277X.2002.00384.x 12270016

[11] Sallis JF , Floyd MF , Rodríguez DA , Saelens BE . The role of built environments in physical activity, obesity, and cardiovascular disease. *Circulation.* 2012;125(5):729–737. 10.1161/CIRCULATIONAHA.110.969022 22311885 PMC3315587

[12] Dusetzina SB , Huskamp HA , Rothman RL , Pinheiro LC , Roberts AW , Shah ND , . Many Medicare beneficiaries do not fill high-price specialty drug prescriptions. *Health Aff (Millwood).* 2022;41(4):487–496. 10.1377/hlthaff.2021.01742 35377748

[13] Dusetzina SB , Besaw RJ , Whitmore CC , Mattingly TJ II , Sinaiko AD , Keating NL , . Cost-related medication nonadherence and desire for medication cost information among adults aged 65 years and older in the US in 2022. *JAMA Netw Open.* 2023;6(5):e2314211. 10.1001/jamanetworkopen.2023.14211 37200029 PMC10196872

[14] Kolak M , Bhatt J , Park YH , Padrón NA , Molefe A . Quantification of neighborhood-level social determinants of health in the continental United States. *JAMA Netw Open.* 2020;3(1):e1919928. 10.1001/jamanetworkopen.2019.19928 31995211 PMC6991288

[15] Centers for Disease Control and Prevention. Behavioral Risk Factor Surveillance System. Survey Data & Documentation. Published 2019. Accessed January 15, 2024. https://www.cdc.gov/brfss/data_documentation/index.htm

[16] Centers for Disease Control and Prevention. PLACES: Local Data for Better Health: Methodology. Published December 8, 2020. Accessed January 21, 2024. https://www.cdc.gov/places/methodology/index.html

[17] US Census Bureau. About the American Community Survey. Published September 26, 2019. Accessed January 22, 2024. https://www.census.gov/programs-surveys/acs/about.html

[18] AHA Annual Survey Database. AHA Data. Accessed January 15, 2022. https://www.ahadata.com/aha-annual-survey-database

[19] Centers for Medicare & Medicaid Services. Data.CMS.gov. Provider of Services File – Hospital & Non-Hospital Facilities. Accessed January 15, 2022. https://data.cms.gov/provider-characteristics/hospitals-and-other-facilities/provider-of-services-file-hospital-non-hospital-facilities

[20] US Department of Agriculture. Service area map datasets. Accessed January 15, 2024. https://www.usda.gov/reconnect/service-area-map-datasets

[21] Chapel JM , Ritchey MD , Zhang D , Wang G . Prevalence and medical costs of chronic diseases among adult Medicaid beneficiaries. *Am J Prev Med.* 2017;53(6S2):S143–S154. 10.1016/j.amepre.2017.07.019 29153115 PMC5798200

[22] US Department of Health and Human Services. Healthy People 2030. Social determinants of health. Accessed July 7, 2022. https://health.gov/healthypeople/priority-areas/social-determinants-health

[23] Oates GR , Jackson BE , Partridge EE , Singh KP , Fouad MN , Bae S . Sociodemographic patterns of chronic disease: how the Mid-South Region compares to the rest of the country. *Am J Prev Med.* 2017;52(1S1):S31–S39. 10.1016/j.amepre.2016.09.004 27989290 PMC5171223

[24] Churchwell K , Elkind MSV , Benjamin RM , Carson AP , Chang EK , Lawrence W , ; American Heart Association. Call to action: structural racism as a fundamental driver of health disparities: a presidential advisory from the American Heart Association. *Circulation.* 2020;142(24):e454–e468. 10.1161/CIR.0000000000000936 33170755

[25] Benavidez GA , Zgodic A , Zahnd WE , Eberth JM . Disparities in meeting USPSTF breast, cervical, and colorectal cancer screening guidelines among women in the United States. *Prev Chronic Dis.* 2021;18:E37. 10.5888/pcd18.200315 33856975 PMC8051853

[26] Gaskin DJ , Thorpe RJ Jr , McGinty EE , Bower K , Rohde C , Young JH , . Disparities in diabetes: the nexus of race, poverty, and place. *Am J Public Health.* 2014;104(11):2147–2155. 10.2105/AJPH.2013.301420 24228660 PMC4021012

[27] Conway D , Li CQ , Wolch J , Kahle C , Jerrett M . A spatial autocorrelation approach for examining the effects of urban greenspace on residential property values. *J Real Estate Finan Econ,* 2008:41150–169. Accessed July 7, 2022. https://link.springer.com/article/10.1007/s11146-008-9159-6

[28] Reynolds R , Dennis S , Hasan I , Slewa J , Chen W , Tian D , . A systematic review of chronic disease management interventions in primary care. *BMC Fam Pract.* 2018;19(1):11. 10.1186/s12875-017-0692-3 29316889 PMC5759778

[29] Wanni Arachchige Dona S , Angeles MR , Hall N , Watts JJ , Peeters A , Hensher M . Impacts of chronic disease prevention programs implemented by private health insurers: a systematic review. *BMC Health Serv Res.* 2021;21(1):1222. 10.1186/s12913-021-07212-7 34763676 PMC8582197

[30] Planey AM , Planey DA , Wong S , McLafferty SL , Ko MJ . Structural factors and racial/ethnic inequities in travel times to acute care hospitals in the rural US South, 2007–2018. *Milbank Q.* 2023;101(3):922–974. 10.1111/1468-0009.12655 37190885 PMC10509521

[31] Zhang X , Bullard KM , Gregg EW , Beckles GL , Williams DE , Barker LE , . Access to health care and control of ABCs of diabetes. *Diabetes Care.* 2012;35(7):1566–1571. 10.2337/dc12-0081 22522664 PMC3379598

[32] Nicholl J , West J , Goodacre S , Turner J . The relationship between distance to hospital and patient mortality in emergencies: an observational study. *Emerg Med J.* 2007;24(9):665–668. 10.1136/emj.2007.047654 17711952 PMC2464671

[33] Levine DA , Kiefe CI , Howard G , Howard VJ , Williams OD , Allison JJ . Reduced medication access: a marker for vulnerability in US stroke survivors. *Stroke.* 2007;38(5):1557–1564. 10.1161/STROKEAHA.106.478545 17395861

